# Enteroviral Transverse Myelitis Presenting as Acute Ataxia in Children: A Case Series

**DOI:** 10.3390/biomedicines13061492

**Published:** 2025-06-18

**Authors:** Luka Švitek, Dominik Ljubas, Nina Krajcar, Maja Vrdoljak Pažur, Ana Tripalo Batoš, Irena Tabain, Srđan Roglić, Lorna Stemberger Marić

**Affiliations:** 1 Department of Infectology and Dermatovenerology, Faculty of Medicine Osijek, J. J. Strossmayer University of Osijek, 31000 Osijek, Croatia; 2 Clinic for Infectious Diseases, University Hospital Centre Osijek, 31000 Osijek, Croatia; 3 Department of Pediatric Infectious Diseases, University Hospital for Infectious Diseases, 10000 Zagreb, Croatia; 4 Department of Infectious Diseases, School of Medicine, University of Zagreb, 10000 Zagreb, Croatia; 5 Department of Paediatric Radiology, Children’s Hospital Zagreb, 10000 Zagreb, Croatia; abatosh@gmail.com; 6 Department of Virology, Croatian Institute of Public Health, 10000 Zagreb, Croatia; irena.tabain@hzjz.hr

**Keywords:** ataxia, enterovirus, infections, myelitis transverse, pediatrics

## Abstract

**Background**: Enteroviruses, members of the *Picornaviridae* family, typically cause asymptomatic or mild infections. However, they can also result in central nervous system (CNS) involvement, with transverse myelitis (TM) occurring only on rare occasions. TM is a syndrome characterized by acute or subacute spinal cord dysfunction, leading to neurological deficits below the level of the lesion. **Case report**: We report a case series of eight pediatric patients admitted over a three-month period, June to August 2024. All patients presented with ataxia and/or other neurological symptoms, alongside abnormal cerebrospinal fluid (CSF) findings. Although ataxia is commonly associated with cerebellitis, magnetic resonance imaging (MRI) in this cohort revealed findings consistent with TM. Notably, all patients demonstrated similar MRI abnormalities. The onset of symptoms occurred over a short time during an enterovirus epidemic. Enteroviral RNA was detected, or the virus was isolated in seven patients, while one patient had a close epidemiological link to the virus. All patients achieved full recovery following immunomodulatory therapy. **Conclusions**: This case series underscores that ataxia may be an atypical symptom associated with TM. Furthermore, there was a notable distinction between the clinical presentation and neuroradiological findings. Immunomodulatory therapy with immunoglobulins and corticosteroids has been shown to be effective and safe, supporting the hypothesis of an immune-mediated pathogenesis in these patients.

## 1. Introduction

Enteroviruses, members of the *Picornaviridae* family, cause infections across all age groups, with young children being the most commonly affected [1,2]. The primary route of transmission is fecal–oral; however, during outbreaks, transmission through swimming pools has also been reported. Currently, approximately 75 enterovirus serotypes are known, causing a wide range of infections that vary from asymptomatic cases to symptomatic conditions, including non-specific febrile illness, herpangina and, in some cases, central nervous system (CNS) infections [1,2,3]. Enteroviral CNS infections are typically mild, most are predominantly presenting as aseptic meningitis in children, particularly during the summer and autumn months [2]. Nonetheless, various other neurological manifestations have also been documented [1,2].

Transverse myelitis (TM) is a syndrome of acute or subacute spinal cord dysfunction that causes neurological impairment below the level of the lesion, usually resulting in motor, sensory, or autonomic nervous dysfunction [4,5]. Since TM represents spinal cord inflammation, ataxia is not expected, as the cerebellum is not affected. The etiology of TM can vary, and it includes infectious causes, autoimmune disorders, intoxications, and paraneoplastic syndrome. In some patients the etiology remains undiscovered, and they are classified as idiopathic [4,5].

In this case series, we present a group of eight pediatric patients diagnosed with TM, hospitalized over a three-month period, June to August 2024. All of these patients exhibited ataxia and other neurological symptoms, along with abnormal cerebrospinal fluid (CSF) findings. The only relevant identified etiology was enteroviral infection.

Written informed consent for inclusion in this case series was obtained from the legal guardians of the patients.

## 2. Case Presentations

### 2.1. Patient 1

A previously healthy 2-year-and-3-month-old female was hospitalized on the third day of a febrile illness that initially presented with vomiting and a rash on her arms and gluteal region. By the second day, she developed arm twitching and neck pain. Her condition worsened on day three, with difficulty walking and ataxia. She had no respiratory or urinary symptoms. Additional clinical information, including performed tests and treatments, is provided in Table 1.

With the treatment, her symptoms gradually resolved, and by discharge she was walking normally, without instability, was fully conscious, and exhibited no neurological deficits.

### 2.2. Patients 2 and 3

Patient 2, aged 2 years and 8 months, and her one-year younger sister, Patient 3, both previously healthy, presented with nearly identical symptoms and illness progression. Initially, both developed fever, headache, and vomiting, and by the second day were ataxic with muscle twitches. Neither patient exhibited respiratory, gastrointestinal, or urinary symptoms, or rash upon admission.

In Patient 2 clinical and neurological improvement was observed from day 7 of the treatment (Table 1), although transient behavioral issues, including episodes of screaming fits, occasional tongue protrusion, and inappropriate laughter, were still noted. All symptoms were resolved by discharge.

Patient 3 developed a mild maculopapular rash that spontaneously resolved during hospitalization. She also showed gradual improvement and regained stable neurological function by the time of discharge.

### 2.3. Patient 4

A previously healthy 3-year-old female patient presented with low-grade fever, malaise, and bilateral knee pain. She complained of not being able to urinate. From the second day of the illness, she began to exhibit walking instability and ataxia. There were no respiratory or gastrointestinal symptoms. Her older sister had symptoms of hand-foot-and-mouth disease a few days prior to the patient’s disease onset, while her second older sister, who had a febrile illness and rash, tested positive for enterovirus (RT-PCR, nasopharyngeal swab).

The patient was treated as shown in Table 1, and urinary catheter was placed due to urinary retention. Over the following days, clinical improvement was noted. One week later, the urinary catheter was removed, and she resumed normal voiding. The patient completely recovered and was discharged home.

### 2.4. Patient 5

A 2-year-and-9-month-old, previously healthy boy had fever and vomited, after which his fever waned, but by the third day of the illness, he started experiencing walking instability.

The management is explained in Table 1. Following combined treatment, the ataxia subsided, and the patient recovered completely.

### 2.5. Patient 6

Patient 6 was an otherwise healthy, 3-years-and-9-month-old female who developed fever and pharyngitis, accompanied by high levels of C-reactive protein (109 mg/dL), and ataxia on the first day of illness. The following day, she experienced an episode of generalized epileptic seizure, accompanied by myoclonic movements of upper extremities and trunk with fixed gaze. Subsequently, she had two similar seizures.

The patient was treated as shown in Table 1. During hospitalization, she expressed intermittent left arm twitching alongside ipsilateral mild muscle weakness. By discharge, complete regression of neurological symptoms was observed.

### 2.6. Patient 7

Patient 7 was an otherwise healthy 3-year-old boy whose first symptoms were headache and photophobia. Afterward he became febrile, and on the fourth day of illness, his mother noticed muscle twitching during sleep, along with ataxia, tremor, and brief episodes of staring and unresponsivenesst accompanied by atypical eye movements throughout the day.

With the treatment (Table 1), ataxia and tremor gradually improved, along with the complete regression of other neurological symptoms.

### 2.7. Patient 8

A previously healthy 4-year-old boy, residing in Croatia at the time of illness but originally from Germany, was admitted to our department with muscle twitches in both upper and lower extremities, accompanied by photophobia. His symptoms had begun three days prior to admission, with fever and vomiting. On examination, the patient presented with ataxia, tremor, and hyper-reflexia in the lower extremities.

Once the treatment (Table 1) commenced, neurological symptoms were resolved. At discharge, he exhibited only very mild instability during fast walking. Since the patient resides abroad, no follow-up was conducted, and it remains unclear whether a full recovery was achieved. However, based on the disease course, a full recovery is anticipated.

## 3. Discussion

**Figure 1:**
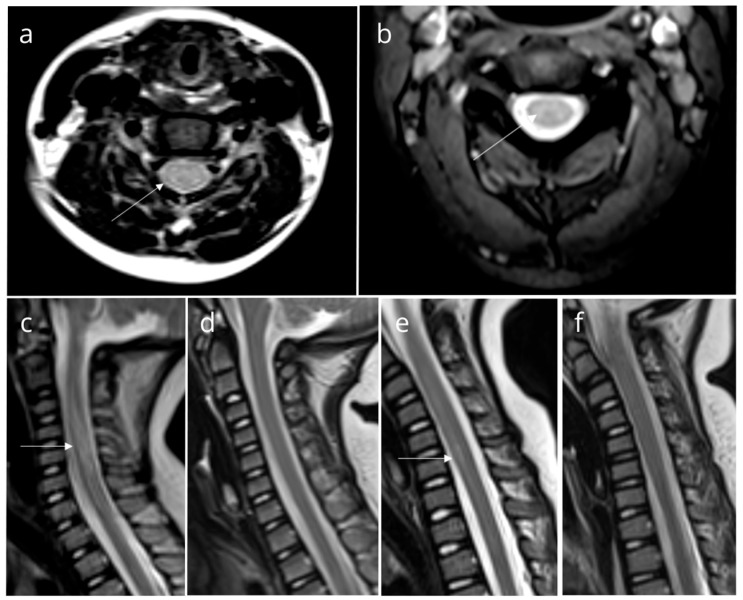
Magnetic resonance imaging (MRI) findings in patients. Axial (**a**) and sagittal (**c**) T2-weighted MRI of Patient 2, axial T2-weighted MRI of Patient 3 (**b**), and sagittal T2-weighted MRI of Patient 6 (**e**) show high-intensity signals suggestive of myelitis (white arrow). Follow-up images of Patient 2 (**d**) and Patient 6 (**f**) reveal normal findings. All other patients had similar findings.

We presented a group of eight pediatric patients with ataxia who were hospitalized over a three-month summer period. Although ataxia is commonly seen in the context of cerebellitis, magnetic resonance imaging (MRI) scans of these patients revealed findings consistent with TM (Figure 1).

To our knowledge, ataxia has not yet been described or observed as a symptom of TM. However, cerebellar ataxia can occur without visible MRI findings in the early stages or in milder forms of cerebellitis, presenting symptoms such as gait instability and discoordination due to cerebellar dysfunction [6]. Moreover, asymptomatic cases of TM and TM cases with silent brain lesions have been reported [7].

Based on these observations, we propose a few possible explanations for the observed discrepancy between clinical presentation and MRI findings in our patient group.

The patients might have experienced asymptomatic TM as an incidental MRI finding. With the increased accessibility and early utilization of MRI in modern medical practice, similar cases may be reported more frequently in the future. Asymptomatic TM possibly may occur not only in autoimmune diseases, as previously described, but also in association with infectious etiologies [7]. Future case reports or prospective observational cohort studies could help confirm or refute this hypothesis.

A demyelinating process affecting the spinal cord, particularly the spinocerebellar and proprioceptive tracts within the lateral and posterior columns, could explain the symptoms of ataxia without directly involving the cerebellum. This pattern is observed in certain spinocerebellar degeneration syndromes, though it has not yet been documented in association with infectious etiologies. Furthermore, degeneration in the brainstem, particularly in the pontine and olivary nuclei, may result in postural instability [8]. Notably, some patients in our cohort exhibited pontine lesions. It is plausible that a similar pathological mechanism could occur in the context of infectious or post- / parainfectious process affecting the spinal cord and pons.

A third consideration is that the MRI was performed early in the course of the illness, and, therefore, cerebellar impairment was not yet visible. However, considering the relatively short recovery period in described patients, this possibility seems unlikely.

Enteroviruses, especially Enterovirus A71, have been reported as a possible cause of TM [5,9]. There is also emerging evidence that other enteroviruses, including Echovirus 18, Coxsackievirus B2, and B5, can cause TM among adolescents and adults [10,11]. In all of the reports good outcomes were achieved by corticosteroids, IVIg, or plasmapheresis clearly implying that enteroviruses trigger immune response during illness, thus contributing to inflammatory process in CNS.

Moreover, enteroviruses have been linked to cerebellar ataxia [2]. Ataxia is not typically associated with TM. Therefore, ataxia as a leading symptom in the discussed cases is unusual and noteworthy.

Considering the etiology in our patient group, two of the observed patients were diagnosed with a definite enteroviral CNS infection, confirmed by a positive RT-PCR for enterovirus in the CSF. Five were classified as having probable enteroviral CNS infection, based on RT-PCR of a pharyngeal swab positive for enterovirus or enterovirus isolated from a stool sample, with all other relevant tests returning negative. None of the patients had positive RT-PCR from the blood sample. Respiratory, stool, blood, and CSF samples are usually used for enterovirus RT-PCR testing and isolation in individuals with neurological symptoms [12]. In one of the observed patients (Patient 4), we were unable to detect viral RNA or isolate the virus. However, the patient’s sisters experienced episodes of febrile illness: one was clinically diagnosed with hand-foot-and-mouth disease, while the other had a rash. To explore a potential epidemiological link between their symptoms, we collected throat swabs from the second sister (as she was accompanying the patient) for RT-PCR testing for enterovirus, which came back positive.

In addition to evaluating for enteroviruses, an extensive search for other possible etiologies of these CNS infections was conducted in all of the patients. This included CSF cultures, RT-PCR of CSF for *Herpesviridae* and bacterial agents, other serum and CSF serology testing, and RT-PCR for arboviral infections and borreliosis, as well as autoimmune panels. In all of the patients reported, empirical acyclovir therapy was initiated and continued until negative RT-PCR for HSV-1, HSV-2, and VZV from CSF were obtained. After comprehensive microbiological studies were performed, no other causative agents were identified.

Therapeutic interventions for TM mostly rely on disease pathophysiology, as TM is primarily an immune-mediated, inflammatory condition, although exact consensus regarding treatment options has not been yet established [5,13]. All of our patients were initially treated with IVIg, followed by pulse doses of corticosteroids if clinical improvement failed to occur. However, a study conducted by Greenberg et al. involving 90 patients concluded that IVIg does not offer superior efficacy compared to corticosteroid treatment [14]. Plasmapheresis is a feasible option in patients suffering demyelinating disorders of CNS in whom improvement with pulse doses of corticosteroids or IVIg is not achieved [15]. In our case series, plasmapheresis was used in only one patient.

All of our patients fully recovered and had no neurologic sequelae on follow-up. The median length of hospitalization was 10 days, with a minimum of 7 days and a maximum of 15 days. Additionally, all follow-up MRI scans conducted at approximately three-month intervals demonstrated the resolution of previously detected lesions. The exception is Patient 8, whom we were unable to follow up due to geographical reasons, but given the disease course, it is expected that full recovery should be achieved. The current literature is scarce on outcomes in pediatric patients diagnosed with TM, but it appears only a minority of patients experience a poor outcome or disease relapse [4,5,16].

## 4. Conclusions

All of the described patients exhibited similar MRI findings and clinical presentations, with the onset occurring over a short time period during an enterovirus epidemic. Furthermore, it is intriguing that various enterovirus subtypes were detected in stool samples, except for Patients 2 and 3, who were siblings (Table 1). Despite this variability, all cases exhibited similar clinical symptoms and MRI findings. It is worth noting that this cluster of eight patients occurred over a relatively short period of three months, after which no similar clinical cases were observed. The reasons for this clustering remain unknown, and future epidemiological and clinical reports might elucidate this phenomenon.

Additionally, this case series is the first to report TM caused by Coxsackievirus B4 and Echovirus 4, as these enteroviruses, to our knowledge, have not been previously identified as causative agents of TM.

This report highlights that ataxia may be an unusual symptom associated with TM. Moreover, the clinical presentation in these cases does not correlate with the neuroradiological work-up. Combined treatment with immunoglobulins and corticosteroids has proven to be a desirable and safe treatment option, as the disease mechanism is likely immune-mediated.

## Figures and Tables

**Table 1:** Demographic and clinical characteristics, laboratory findings, imaging results, treatment, and outcomes in patients.

	Sex	Age	Clinical Presentation	CSF				Enteroviral RNA Detected from (RT-PCR) *			Type of Enterovirus **	MRI Findings	Treatment	Outcome
				WBC [n/µL]	PMN [%]	MNC [%]	Proteins [g/L]	Pharynx	Blood	CSF				
Patient 1	F	2 years and 3 months	Fever, vomiting, rash on the gluteal region and upper extremities, arm twitching, ataxia	235	68	32	0.43	+	−	−	Echovirus 4	Transverse myelitis (C1 to C6), hyperintense signals in posterior pons and medulla oblongata	IVIg (2 g/kg) Methylprednisolone (pulse doses 15 mg/kg/day of for 3 days, followed by taper-off), and plasmapheresis (4 cycle)	Full recovery
Patient 2 ***	F	2 years and 8 months	Fever, vomiting, headache, tremor, ataxia, transient behavioral issues	533	25	75	0.54	+	−	−	Enterovirus 71	Transverse myelitis (C1 to C7)	IVIg (2 g/kg) Methylprednisolone (pulse doses 20 mg/kg/day for 3 days, followed by taper-off)	Full recovery
Patient 3 ***	F	1 year and 8 months	Fever, vomiting, headache, tremor, ataxia, maculopapular rash	1110	70	30	0.63	−	−	+	Enterovirus 71	Transverse myelitis (C4 to C6)	IVIg (1.5 g/kg)	Full recovery
Patient 4	F	3 years and 3 months	Fever, lower extremity and abdominal pain, urinary retention, tremor, ataxia	48	7	93	0.53	−	−	−	−	Transverse myelitis of cervical and thoracic spinal cord	IVIg (2 g/kg) Methylprednisolone (pulse doses 16 mg/kg/day for 3 days, followed by taper-off)	Full recovery
Patient 5	M	2 years and 9 months	Fever, vomiting, ataxia	13	1	99	0.28	+	n/p	−	Coxsackie B4 virus	Transverse myelitis (C2 to C7)	IVIg (1 g/kg) Methylprednisolone (pulse doses 15 mg/kg/day of for 3 days, followed by taper-off)	Full recovery
Patient 6	F	3 years and 9 months	Fever, rash on the abdomen and lower extremities, pharyngitis, generalized epileptic seizures, tremor, ataxia	234	87	23	0.39	+	−	−	−	Transverse myelitis (C1 to C6), encephalitis	IVIg (2 g/kg)	Full recovery
Patient 7	M	3 years and 2 months	Fever, headache, photophobia, fasciculations, focal epileptic seizures, ataxia	129	88	12	0.34	+	n/p	+	Coxsackie B4 virus	Transverse myelitis of the whole spinal cord, encephalitis, hyperintense signals in posterior pons and medulla oblongata	IVIg (2 g/kg)	Full recovery
Patient 8	M	4 years and 3 months	Fever, vomiting, photophobia, abdominal pain, urinary retention, tremor, ataxia	613	8	92	0.49	+	−	−	−	Transverse myelitis of cervical spinal cord, hyperintense signals in medulla oblongata	IVIg (2 g/kg), Methylprednisolone (pulse doses 13 mg/kg/day for 3 days, followed by taper-off)	Recovery with minor neurological impairment

C—cervical vertebra; CSF—cerebrospinal fluid; F—female; IVIg—intravenous immunoglobulin; M—male; MNC—mononuclear cells; MRI—magnetic resonance imaging; n/p—not performed; PMN—polymorphonuclear leukocytes; RT-PCR—reverse transcription polymerase chain reaction; WBC—white blood cells; +—positive; −—negative; * ViroReal^®^ Enterovirus PCR Kit (Ingenetix, Vienna, Austria); ** isolation and identification from fecal samples; *** siblings.

## Data Availability

All data generated and analyzed in the presented. Study are included in this published article.

## Abbreviations

The following abbreviations are used in this manuscript:

CNS	Central nervous system
CSF	Cerebrospinal fluid
HSV	Herpes simplex virus
IVIg	Intravenous immunoglobulin
MRI	Magnetic resonance imaging
RNA	Ribonucleic acid
RT-PCR	Reverse transcription polymerase chain reaction
TM	Transverse myelitis
VZV	Varicella zoster virus
